# Multiplexed expression and screening for recombinant protein production in mammalian cells

**DOI:** 10.1186/1472-6750-6-49

**Published:** 2006-12-22

**Authors:** Susan DJ Chapple, Anna M Crofts, S Paul Shadbolt, John McCafferty, Michael R Dyson

**Affiliations:** 1 The Atlas of Protein Expression Project, The Wellcome Trust Sanger Institute, Wellcome Trust Genome Campus, Hinxton, Cambridge, CB10 1SA, UK

## Abstract

**Background:**

A variety of approaches to understanding protein structure and function require production of recombinant protein. Mammalian based expression systems have advantages over bacterial systems for certain classes of protein but can be slower and more laborious. Thus the availability of a simple system for production and rapid screening of constructs or conditions for mammalian expression would be of great benefit. To this end we have coupled an efficient recombinant protein production system based on transient transfection in HEK-293 EBNA1 (HEK-293E) suspension cells with a dot blot method allowing pre-screening of proteins expressed in cells in a high throughput manner.

**Results:**

A nested PCR approach was used to clone 21 extracellular domains of mouse receptors as CD4 fusions within a mammalian GATEWAY expression vector system. Following transient transfection, HEK-293E cells grown in 2 ml cultures in 24-deep well blocks showed similar growth kinetics, viability and recombinant protein expression profiles, to those grown in 50 ml shake flask cultures as judged by western blotting. Following optimisation, fluorescent dot blot analysis of transfection supernatants was shown to be a rapid method for analysing protein expression yielding similar results as western blot analysis. Addition of urea enhanced the binding of glycoproteins to a nitrocellulose membrane. A good correlation was observed between the results of a plate based small scale transient transfection dot blot pre-screen and successful purification of proteins expressed at the 50 ml scale.

**Conclusion:**

The combination of small scale multi-well plate culture and dot blotting described here will allow the multiplex analysis of different mammalian expression experiments enabling a faster identification of high yield expression constructs or conditions prior to large scale protein production. The methods for parallel GATEWAY cloning and expression of multiple constructs in cell culture will also be useful for applications such as the generation of receptor protein microarrays.

## Background

Functional genomic applications have increased the requirement for producing large protein sets including the generation of protein microarrays for mapping protein-protein, nucleic acid or small molecule interactions [1,2], high throughput antibody generation [3,4] and structural genomics [5]. Although expression of recombinant proteins in bacteria is widely used, mammalian expression systems have advantages for the production of mammalian proteins, allowing correct folding or authentic post-translational modifications. The use of transient expression systems rather than stable expression systems has facilitated the rapid production of cells producing proteins of interest. There are in fact many examples where large scale transient transfection of HEK-293E cells have successfully been used to produce proteins [6-10]. In our laboratory, 50 – 200 ml transient transfections of HEK-293E cells routinely provide 10–1000's μg amounts of secreted protein fragments of receptor extracellular (EC) domains (unpublished). A proportion of attempted transient transfections however result in the absence of secreted protein. The ability to pre-screen multiple expression vectors for secreted protein production on a small scale and thus identify failures prior to the larger scale transfections would reduce time, cost of reagents and allow an increased number of proteins to be produced at the large scale. The method would also allow the rapid screening of different expression conditions to optimise expression including media formulation, co-expression of chaperones [11], anti-apoptotic proteins [12] or binding partners [13]. The effect of fusion partners, signal peptide sequences or truncations could also be rapidly assessed. This is particularly important during the identification of stable cell lines yielding high levels of therapeutic antibodies or proteins.

The use of suspension cells rather than adherent cells [8,9,14,15] for a transient transfection pre-screen reduces the time required to perform the pre-screen and lends itself to direct comparison of subsequent suspension cell growth in the 50 – 200 ml shake flask cultures. The advantage of 24 well blocks for transient transfections is that this allows the multiple processing of expression vectors facilitating the rapid detection of expression failures. The aim of this work was to devise and implement a system for pre-screening expression vectors for positive hits prior to large scale protein production work. Here we show that small scale transient transfection of mammalian suspension cells together with analysis by dot blot can be used to assess positive expression hits in a multiparallel high throughput manner.

## Results

### Vector construction

**Figure 1:**
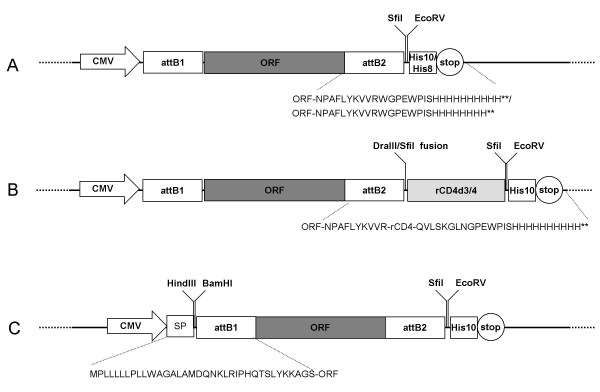
**Expression vector constructs used in transient transfections and purified protein controls**. (A) Schematic representation of His10 and His 8 tagged expression vectors with amino acid sequence of attB2 site and His tag shown. (B) Schematic representation of rCD4(d3+4)-His10 tagged expression vector used to express mouse test proteins, amino acid sequence from attB2 site to stop codon (rCD4 sequence has been represented as rCD4) is shown. (C) Schematic representation of His10 tagged expression vector containing signal peptide (SP) at N terminus with amino acid sequence of SP and attB1 site shown. CMV = CMV promoter element, ORF = open reading frame, attB1 = attB1 recombination site, attB2 = attB2 recombination site, rCD4 = rat CD4 domains 3 and 4 tag, His10 = decahistidine tag, His8 = octahistidine tag, stop = stop codon and SP = CD33 signal peptide sequence.

GATEWAY cloning technology (Invitrogen) was chosen for the generation of expression vectors making it possible to rapidly generate vectors containing the same coding sequence with different vector backbone options (for example N or C-terminal tags, different expression vector cassettes). 26 proteins representing the extracellular domains of receptors (including four proteins used as controls, rCD4, mCD4, mCD200-rCD4 and EfnB2, previously shown to express well in the HEK-293E transient transfection system (data not shown)) were chosen for this small scale transient transfection pre-screen (Table 1). All are mouse proteins with the exception of the rat CD4 control protein (rCD4). All vectors with the exception of the three control proteins contained a C-terminal rCD4 (domains 3 + 4)-His10 tag. Brown and Barclay, 1994 [16] have previously shown that fusion of the extracellular domains of receptors to rCD4 (domains 3 + 4) can both enhance their expression and produce the proteins in a monomeric form suitable for kinetic and affinity analysis. Secretion of the tagged test proteins into the culture supernatant was driven by the native signal peptide. The list includes proteins with different numbers of Ig domains (between 1–4) and N-glycosylation sites (between 0–8). The genes were isolated from cDNA libraries and cloned into destination vectors (Figure 1) using GATEWAY recombinational cloning (this cloning method has been previously described by Hartley *et al*., 2000 [17]). The destination vector used in this study was constructed by modifying the pTT3 vector [7]. The pTT3 vector backbone features the Epstein-Barr virus (EBV) oriP allowing episomal replication of the plasmid within transfected cells and an improved human cytomegalovirus (CMV) expression cassette containing an intron downstream from the promoter serving to enhance expression [7]. Figure 1 shows schematic of expression vector constructs for both test and control vectors (see Methods).

### Growth kinetics of HEK-293E cells in 24 deep well blocks

To identify the seeding densities allowing survival and growth of HEK-293E cells in 24 well blocks (Qiagen), cells were seeded at 2.5 × 10^5^, 5 × 10^5 ^or 1 × 10^6 ^cells/ml in a 4 ml culture volume. The highest viability and consistent logarithmic growth was identified at seeding densities of 5 × 10^5 ^and 1 × 10^6 ^cells/ml, which produced very similar growth kinetics. Cells seeded at 2.5 × 10^5 ^cells/ml showed low growth and poor viability from 24 hours post seeding (data not shown). The rotation speed at which the cells were grown was found to be optimal at 400 rpm (orbital throw 3 mm) and cells did not stay in suspension when speeds of 250 – 350 rpm were tested (data not shown). The addition of 0.1 % pluronic, a non-ionic detergent, allowed cells to continue to grow beyond 48 hours post seeding, presumably due to a reduction in shear stress (data not shown).

**Figure 2:**
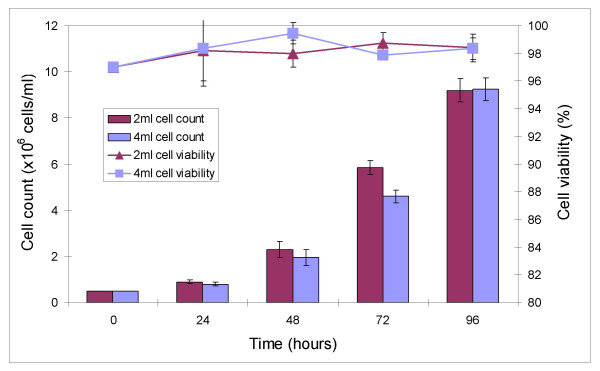
**Comparison of HEK-293E cell growth in different volumes within a 24 well block**. Cells were seeded at 0.5 × 10^6 ^cells/ml in either 2 ml or 4 ml. Cells were analysed in duplicate and counted using a Neubauer haemocytometer to calculate cell growth and viability.

The use of smaller volumes reduces both the number of cells in culture and the amount of DNA required for their transient transfection. Two culture volumes were tested, 2 ml and 4 ml, with cells seeded at 5 × 10^5 ^cells/ml in both. Cell counts and cell viability were monitored from duplicate wells every 24 hours over a 96 hour period. We analysed samples in triplicate using trypan blue exclusion dye to assess cell viability. Cell growth and viability in 2 ml culture volume closely matched that of the 4 ml culture volume Figure 2). Cell viability remained above 97 % in both culture volumes (Figure 2 line graph), cells doubled approximately every 24 hours in both culture volumes (Figure 2 bar chart). All subsequent work was done using the 2 ml culture volume.

### Comparison of growth kinetics of HEK-293E cells grown in block culture and 50 ml shake culture

**Figure 3:**
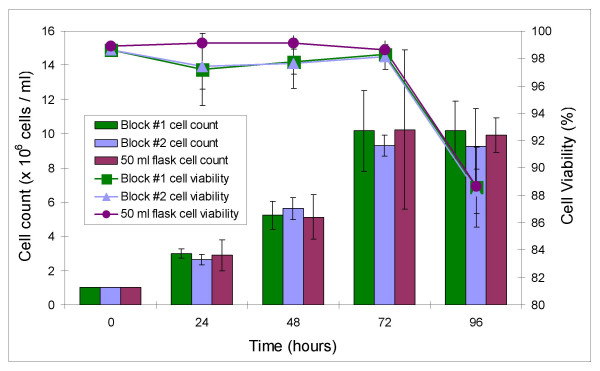
**Comparison of HEK-293E growth kinetics in 24 well blocks culture and 50 ml flask culture**. Cells were seeded at 1 × 10^6 ^cells/ml in either a total of 50 ml (flask) or 2 ml (24 well blocks). Two separate 24 well blocks were analysed alongside one large scale flask culture in triplicate. Cells were counted using a Neubauer haemocytometer to calculate cell growth and cell viability. Error bars represent standard deviations calculated from the data (n = 3).

The growth kinetics of cells grown in 2 ml culture volume in 24 well blocks with cells grown in 50 ml shake culture was compared. Our standard seeding density for HEK-293E cells in 50 ml shake culture is 1 × 10^6 ^cells/ml. To maintain consistency, cells were seeded at 1 × 10^6 ^cells/ml in both 2 ml and 50 ml culture volumes and measured cell growth and viability every 24 hours for 96 hours. Samples were analysed in triplicate using trypan blue exclusion dye to assess cell viability. Cell growth characteristics in both the 24 well blocks and shake flask cultures were very similar at all time points analysed (Figure 3). In both the 2 ml 24 well blocks and 50 ml shake culture, from 72 hours post seeding (hps) living cells began to aggregate, making accurate counting more difficult and causing the measurements to vary more than at other time points. By 96 hps, cells, including aggregated cells, are beginning to die and become stained with trypan blue dye. Stained cells are not included in cell growth calculations which results in smaller standard deviations between counts. The reduction in total culture volume does not have an effect on cell growth and viability. In figures 2 and 3 it is clear that when cell culture density reaches approximately 1 × 10^7 ^cells/ml the cell viability starts to decrease. This is observed after 72 hrs (Figure 3) and after 96 hrs (Figure 2) due to the difference between initial seeding densities.

### Equivalent protein expression in small or large scale culture

**Figure 4:**
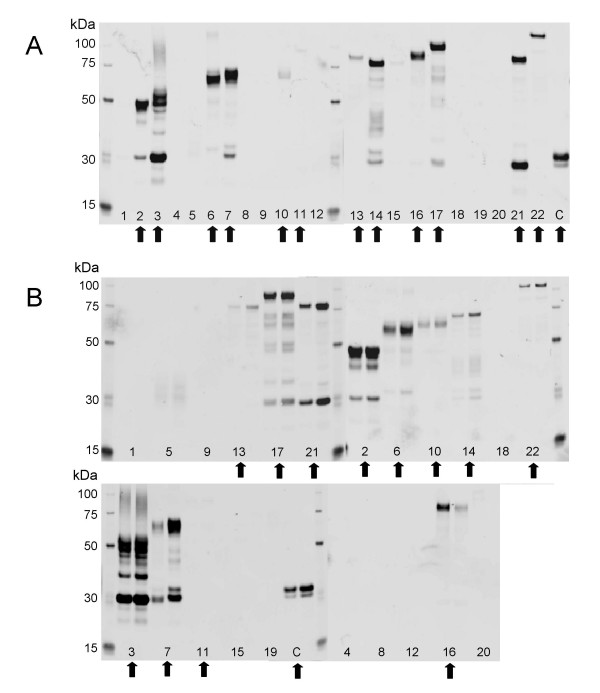
**Protein expression in 24 well blocks and 50 ml flask culture**. Supernatants from 50 ml shake flask (A) and 2 ml 24 well block (B) transient transfections were analysed by Cy5 western blot. The 13 vectors which are positive for protein production are indicated by arrows. In B, the two different seeding density samples from the 24 well blocks were run adjacent to each other with the lower seeding density sample loaded first. The control protein C was rCD4 (domains 3 + 4)-His10 expressed from vector C, Figure 1.

Twenty three different expression vectors were tested for secreted protein production following transient transfection of HEK-293E cells either in large (50 ml) or small (2 ml) scale cultures. 50 ml culture volumes were seeded at 1 × 10^6 ^cells/ml and transfection supernatants were harvested at 120 hours post transfection (hpt) and analysed by fluorescent Cy5 western blot (Figure 4A). Supernatants showed 13 secretion positive vectors (#2, 3, 6, 7, 10, 11, 13, 14, 16, 17, 21, 22 and C) and 10 secretion negative vectors (#1, 4, 5, 8, 9, 12, 15, 18, 19 and 20). Failure to secrete is unlikely to be caused by expression from a GATEWAY vector, which requires the translation of the attB2 linker (NPAFLYKVV) as shown in Figure 1, because a comparison of a GATEWAY and non-GATEWAY expression vector showed similar levels of expression for Cd200 EC domain fused to rCD4 (domains 3 + 4) (data not shown). It is more likely that failure to secrete is due to a property of the protein to be expressed and reasons could include RNA instability, RNA secondary structure resulting in reduced translation levels, inefficient native signal peptides, susceptibility to proteolysis or the requirement of heterodimerisation for stability and secretion. The highest signal intensity was observed for vector #3 and the weakest positive signal intensity for vector #5 (Table 2). Small scale transfections were performed using two different seeding densities (5 × 10^5 ^or 1 × 10^6 ^cells/ml) since both these densities gave good growth kinetics in the 2 ml culture volume. Transfection supernatants were harvested at 120 hpt and analysed by western blot as above. The highest levels of secreted protein were observed when the higher seeding density was used (Figure 4B). Both western blot and signal intensity data confirmed an identical pattern of expression in 2 ml and 50 ml culture volumes, the same vectors were positive and negative for secreted protein in both and the most highly expressed protein (#3) was also the same (Table 2). In most cases supernatants from both transfection culture volumes showed varying amounts of cleaved rCD4 tag at approx 30 kDa. Cleavage was previously observed for expression of a T cell receptor V domain – rCD4 (domains 3 + 4) fusion [16]. The variable levels of cleavage observed in Figure 4, between different receptors, suggest that cleavage is occurring at the C-terminus of the target receptors rather than within the constant attB2 linker – rCD4 polypeptide. Most proteins display a higher observed molecular weight compared with the calculated molecular weight in Table 1 due to glycosylation. In addition, some protein bands appear diffuse. When similar proteins produced in this HEK-293E system are deglycosylated they run as sharper bands on a western blot (data not shown).

### Dot blot conditions

**Figure 5:**
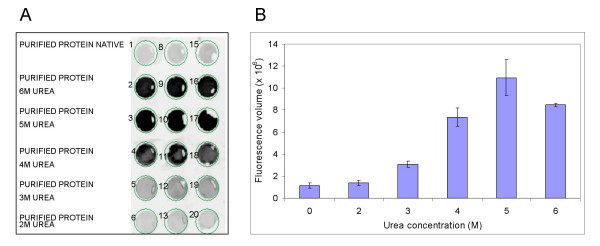
**Determination of dot blot denaturation conditions to use**. Purified EfnB2-EC in mock supernatant was incubated in urea at different concentrations for 1 hour at room temperature prior to loading onto a nitrocellulose membrane in triplicate using the dot blot apparatus. After detection of His tagged proteins (see Methods section) with anti-His tag mAb and Cy5 labelled goat anti-mouse antibody the blots were scanned on a Typhoon 8600 variable mode imager (A) and dots were quantitated using ImageQuant software (B).

Western blot analysis provides essential information about the successful expression of secreted protein but does not lend itself to high-throughput analysis. In order to analyse multiple samples in parallel we investigated the use of the dot blot method to provide much of the same information as western blotting. This involved immobilising proteins onto a nitrocellulose membrane and probing with mouse anti-His-tag and anti-mouse Cy5 labelled antibodies (see Methods). Prior to analysing samples by the dot blot method, various conditions for dotting samples onto nitrocellulose membrane were tested to achieve the best signal. Previous attempts with supernatants and purified protein under native conditions gave uneven binding (data not shown). A purified control protein, EfnB2, diluted in mock supernatant was analysed under native conditions and in varying concentrations (6 M to 2 M) of urea to promote denaturation (Figure 5A). A total of 2.4 μg of protein was loaded per dot and all conditions were analysed in triplicate. The intensity of each dot, measured using ImageQuant software, is represented as a bar chart (Figure 5B). Immobilisation in 2 M urea showed similar levels to the native conditions. The highest signal was observed using 5 M urea and this was chosen as the condition for future dot blot analysis of purified protein and supernatants.

### Dot Blot calibration curves

**Figure 6:**
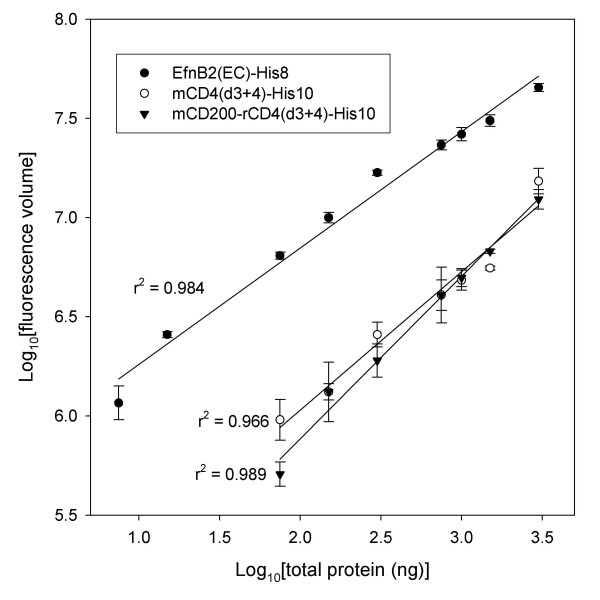
**Dot blot calibration curves using three standard proteins**. Three purified proteins (quantitated by SEC) diluted in mock supernatant and denatured by addition of urea to a final concentration of 5 M were analysed within a range of 0 to 3000 ng per dot by dot blot. R^2 ^were calculated by linear regression analysis (SigmaPlot, SPSS Inc). Error bars represent standard deviations calculated from the data (n = 3).

In order to validate the use of dot intensity to quantitate the amount of expressed protein, calibration curves were made using three purified control proteins, EfnB2(EC)-His8, mCD4(d3 + 4)-His10 and mCD200-rCD4(d3+4)-His10. Proteins were diluted in mock supernatant and urea was added to a final concentration of 5 M. The amount of protein loaded per dot varied from 0–3000 ng and all samples were analysed in triplicate (Figure 6). The intensity of each dot was measured using ImageQuant software and calibration curves plotted. Signal intensity at a given amount of protein varied, suggesting that some proteins bind to the membrane at different efficiencies but each individual protein showed a linear relationship between dot signal intensity and amount of protein per dot over the range of 7.5 ng to 3 μg (25 ng/ml to 10 μg/ml) for EfnB2(EC)-His8 and 75 ng to 3 μg (250 ng/ml to 10 μg/ml) for mCD4(d3+4)-His10 and mCD200-rCD4(d3+4)-His10. Fluorescence detection, despite being 10-fold less sensitive compared to chemiluminscence, gave a superior linear dynamic range [18,19].

### Analysis of the 2 ml transfection supernatants by dot blot

**Figure 7:**
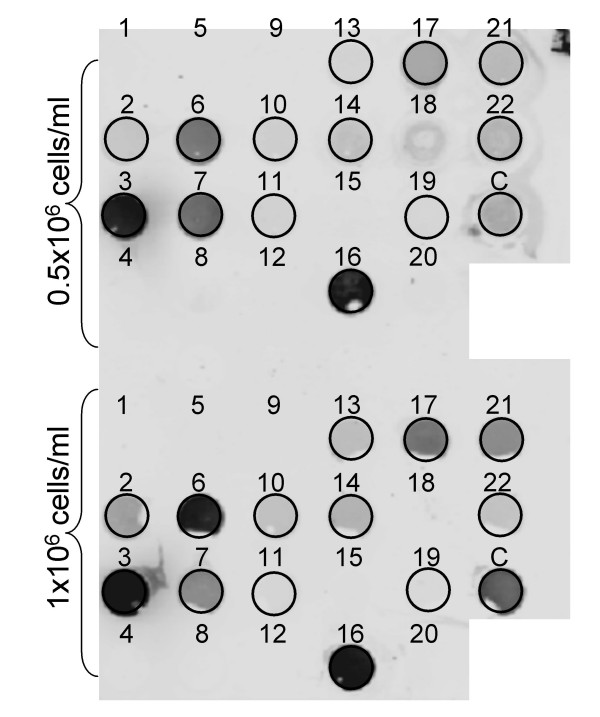
**Screening expression yield of 24 well cultures by dot blot**. Supernatants from 2 ml 24 well blocks transient transfections were analysed by Cy5 dot blot using denaturing conditions of 5 M urea. The top half of the blot represents supernatants from cells seeded at 5 × 10^5 ^cells/ml and the bottom half of the blot represents supernatants from cells seeded at 1 × 10^6 ^cells/ml. Dots showing secretion positive hits are indicated by a circle (14 positive hits and 9 failures). Those dots corresponding to successful purification hits from large scale transient transfections are shown by a square box around the vector number. The signal intensity of the dots is shown in table 2. The control protein C was rCD4 (domains 3 + 4)-His10 expressed from vector C, Figure 1.

Supernatants from the small scale transfections were analysed by fluorescent Cy5 dot blot. Supernatants were diluted in urea to a final concentration of 5 M prior to loading onto the membrane. In Figure 7, the top half of the membrane shows the supernatants from the lower seeding density cultures and the bottom half shows supernatants from the higher seeding density cultures. As with the western blots (Figure 4B) the higher seeding density resulted in signals higher than those seen with the lower seeding density. The dots from the bottom half of the membrane showing a positive signal were analysed using ImageQuant software. There were 14 vectors that showed secreted protein that was detectable by dot blot (2, 3, 6, 7, 10, 11, 13, 14, 16, 17, 19, 21, 22 and C) and 9 vectors that showed no detectable secreted protein expression (1, 4, 5, 8, 9, 12, 15, 18 and 20). Again, vector #3 showed the highest signal intensity, however, the lowest intensity from a secretion positive vector was observed for vector #19 (Table 2).

### Dot blot screen correlates with purified protein yield

The supernatants from the 50 ml culture supernatants were purified by immobilised metal affinity chromatography (IMAC) followed by a desalt step over a sephadex column and cation exchange chromatography to concentrate the protein (see Methods section). The yield of purified product was estimated from the area under the observed 280 nm absorbance peak during the sephadex column step and this was converted to the protein yield in μg using the calculated extinction coefficient for each protein [20]. The calculated yield values are contained in the penultimate column headed "Ex-IMAC AKTA" in Table 2. It was found that the protein yields calculated here were usually over-estimates possibly due to some residual imidazole buffer contributing to the 280 nm absorbance, the shoulder absorbance of the bicine buffer or the presence of protein aggregates. Selected purified proteins were also spun to remove aggregates, analysed by analytical size exclusion chromatography (SEC) (see Methods section) and the calculated yields are shown in the last column headed "analytical SEC" in Table 2. Single peak integration was linear in the range of 40 – 700 ng using maltose binding protein (MBP) and bovine IgG as standards (data not shown). The proteins that showed the highest intensity of signal by dot blot (Figure 7, vectors #17, 21, 2, 6, 14, 3, 7 and control) went on to be successfully purified from the large scale culture (with the exception of the product of vector #16) showing the good predictive value of this system. Vectors #11 and 19 were failures when analysed by western blot (Figure 4) but showed a weak positive hit by dot blot (Table 2). Vectors that showed a small visible peak of protein when run on the AKTA showed a lower intensity on the dot blot. These weakly positive vectors (vector #13, 10, 11, 22, and 16) are good candidates for larger scale (>50 ml) transient transfections to produce increased amounts of protein.

## Discussion

A number of expression systems can be used for the production of recombinant proteins. Mammalian expression systems, unlike the prokaryotic and lower eukaryotic expression systems, are efficient in producing active mammalian proteins due to their post-translational processing machinery and presence of endogenous levels of binding partners that may be necessary to stabilise the expressed protein of interest. In order to achieve a high yield of protein for analysis, mammalian expression systems tend to have been based on isolated, high expression stable cell lines [21]. A disadvantage of this method is the time taken to establish these stable cell lines. The transient mammalian expression system described here [7] has many of the benefits but does not suffer from the time constraints of stable cell line expression systems.

The ability to pre-screen expression vectors to identify those giving successful protein expression prior to large scale protein production helps to reduce time, cost and lends itself to high throughput handling of samples. The use of pre-screening with adherent cell cultures is well practiced but there is little mention in the literature of using suspension cells for pre-screening. Girard *et al*., 2001 [22] reported the use of a twelve-well microtiter plate agitated on a rotational shaker to grow suspension mammalian cells. They tested intracellular GFP expression and found similar expression between small scale cultures and larger cultures grown in spinner flasks or bioreactors. Chambers *et al*., 2004 [23] and Bahia *et al*., 2005 [13] have both described the use of 24 well blocks for the growth of insect cells in suspension and the screening of baculovirus expression constructs. Recently Davies *et al*., 2005 [24] described a method for transient transfection of HEK-293E cells in 24-well blocks with a lipid based transfection reagent and validated this by comparing with scaled up shake-flask expression for a set of intracellularly expressed kinases. We describe the use of 24 well blocks for pre-screening secreted proteins following transient transfection of HEK-293E cells in suspension using PEI. This pre-screen method lends itself to the use of liquid handling robots for dispensing cells and reagents thus speeding up the process further.

This small scale transient transfection method can yield on average 10 μg/ml and so could be used in cases where very small amounts of proteins are sufficient for downstream work (e.g. protein micro-arrays) or could be used as a pre-screen prior to larger scale culture. We found that cells grown in 2 ml cultures in 24 well blocks mimic cell growth and viability of cells grown in 50 ml shake cultures. These 2 ml cell cultures were also successfully transfected with expression vectors using PEI in the 24 well blocks and protein expression profiles matched those of 50 ml transfection cultures. The use of the dot blot method allows the screening of large numbers of samples and can be used in conjunction with the 24 deep well block to allow the screening of 4 × 24 well blocks in parallel.

We found that the addition of urea helps immobilisation of proteins on the membrane. The ability of urea to unfold proteins and expose hydrophobic amino acids may make it easier for the proteins to adhere to the membrane and counteract the presence of hydrophilic glycosylated residues that interfere with binding of the protein to the membrane. *E. coli *MBP, which is not glycosylated, did not require urea denaturation for efficient binding to nitrocellulose (data not shown). The dot-blot method is semi-quantitative when comparing different proteins due to the differential binding ability of various proteins to the membrane. Even when proteins are denatured they bind to the membrane with differing efficiency. This can be seen on the dot blot calibration curves where three control proteins showed binding within a linear range but each protein bound to the membrane with varying success (Figure 6). There appears to be no correlation between efficiency of binding to the membrane and protein size, pI or number of glycosylation sites. However, the use of the dot blot method for quantitating multiple different constructs (for example containing different truncations of a single protein when annotated domain boundary information is absent) could be very useful and the pre-screen and dot blot combination is useful for optimising culture conditions for a single protein.

We found that in almost every case the intensity of dots on the dot blot correlated to success of proteins being purified. The notable exception to this was #16, which despite being strongly positive by dot blot narrowly failed protein purification by falling below the required 200 μg for peak collection. The reason for this has not been determined but might be due to the differential binding of proteins to the membrane as seen with the standard curves. It may also be explained by the lack of accessibility of the his-tag during purification under native conditions. The analysis of small scale pre-screen cultures is not limited to western blot and dot blot analysis. Alternative methods to quantitate small scale expression may include bead capture via the His tag, elution, followed by quantitation of the purified protein or ELISA based screens.

## Conclusion

We have shown that small scale transfection of HEK-293E cells are a scalable way to produce protein and can be performed in 24 deep well blocks correlating well to what is seen in larger scale transfections. The use of dot blot screening of supernatants allows the rapid identification of vectors or conditions yielding sufficient secreted protein to proceed successfully through purification when grown in a larger scale. By identifying failed constructs early, the approach reduces time, cost and labour of processing negative experiments at a large scale.

## Methods

### Construction of C-terminal tagged pTT3 GATEWAY destination vectors

The pTT3 vector was digested with PmeI and BamHI to allow the introduction of a PmeI/BglII digested PCR fragment to create pTT3DestHis10. The PCR fragment consisted of a GATEWAY cassette with 5' PmeI-HindIII and 3' SfiI-EcoRV-His10-BglII-HindIII flanking regions. The GATEWAY cassette was amplified from an in house vector pDest6 using the following forward and reverse primers respectively. 5':TTATTAGTTTAAACAAGCTTAGGATCCCCCATCAAACAAG:3' and 5':TATTATAAGCTTAGATCTCGAATTAGTGATGGTGATGGTG:3'. The resulting pTT3DestHis10 vector contained 5' PmeI-HindIII-GATEWAY cassette-SfiI-EcoRV-His10-Bam/Bgl fusion 3'. Vector pTT3DestHis10 was further modified by addition of a tag, rCD4 domains 3 and 4, at the SfiI site. The rCD4 tag was introduced as a 5' DraIII-rCD4-SfiI 3' insert to create pTT3DestrCD4(d3+4)-His10. The rCD4 tag was obtained from a vector supplied by Neil Barclay using the following forward and reverse primers respectively. 5': TACACGAAGTGACATCCATCACGGCCTATAAGAGTG:3' and 5':TAGGCCATTCTGGCCCATTCAACCCTTTGGATAAAACCTGG:3'. Vector pTT3DestSPHis10 was constructed by introducing the CD33 signal peptide sequence into pTT3DestHis10 at the PmeI site as a phosphorylated blunt linker fragment. The forward and reverse oligos used are shown respectively: 5':ACCATGCCGCTGCTGCTACTGCTGCCCCTGCTGTGGGCAGGGGCCCTGGCTATGGATCA:3'

5':TGATCCATAGCCAGGGCCCCTGCCCACAGCAGGGGCAGCAGTAGCAGCAGCGGCATGGT:3'

### cDNA isolation and expression vector generation

A nested PCR strategy was used to isolate protein encoding ORFs directly from cDNA [25] and adapted for GATEWAY cloning. Briefly 2 sets of primer pairs were designed, the first pair of optimised primers binding 1 – 200 bp 5' and 3' of the ORF using in house software and a second set of forced primers targeted to the beginning (start Met) and end (5 aa upstream of TM domain) of the ORF. All primers were designed with melting temperatures around 60°C. PCRs were carried out as described [18] with the following additions. For entry clones to be transferred to C-terminal tag expression vectors the PCR-2 forward and reverse primers used were 5' AAAAAGCAGGCTACC 3' and 5' AGAAAGCTGGGTT 3' respectively with the forward primer encoding the kozak sequence. The recombinational cloning of attB flanked PCR products with an attP containing pDONR vector to generate a set of entry plasmids was as described previously [18]. The LR recombination reactions using sequence confirmed entry vector and pTT3 adapted destination vector to generate expression vectors [18] were used to transform E. coli DH5α cells (Invitrogen). Vector pTT3DestHis10 was used to generate the control vector expressing mouse CD4 domains 3 + 4 (Figure 1A). Vector pTT3DestrCD4(d3+4)-His10 was used to generate all the test vectors used in this study (Figure 1B). Vector pTT3DestSPHis10 was used to generate the control vector expressing rat CD4 domains 3 + 4 (Figure 1C). A similar vector was used to generate the control vector expressing mouse EfnB2 except the C-terminal tag was 8xHis residues rather than 10. Plasmid DNA for use in transfections of HEK-293E cells was prepared by either maxi-prep filter kit (Invitrogen) or mini-prep kit (Qiagen). DNA was quantitated using the picogreen assay (Molecular probes).

### Cell culture

Suspension adapted HEK-293E cells were originally obtained from Yves Durocher (Biotechnology Research Institute, National Research Council Canada, Montreal, Canada). HEK-293E cells were maintained in FREESTYLE media (Invitrogen) supplemented with 1 % FBS (Invitrogen) and 50 μg/ml G418 (Novagen). Cells were maintained in agitated vented Erlenmeyer flasks (Corning) at 120 rpm (orbital throw 25 mm), 37°C, 5 % CO_2 _and 40 % humidity. Cells were routinely maintained at > 95 % viability and passaged when the cell density reached 1–4 × 10^6 ^cells/ml. For cells grown in 24 well blocks 0.1 % pluronic (Sigma) was also added. For culture volume comparison in 24 well blocks, HEK-293E cells were seeded in 24 well blocks at 0.5 × 10^6 ^cells/ml in a total volume of 2 or 4 ml. Blocks were sealed with an air-pore membrane and incubated in an Infors Multitron II incubator at 37°C, 5 % CO_2_, 40 % humidity at 400 rpm. Samples were analysed every 24 hours over a period of 96 hours for cell growth and viability. For the growth kinetic studies cells were seeded at 1 × 10^6 ^cells/ml in a total volume of 50 ml (flask) or 2 ml (24 well blocks) and grown as above.

### Transient transfections

Large scale transient transfections were performed where HEK-293E cells were seeded at 5 × 10^5 ^cells/ml in 50 ml and incubated for 24 hours prior to transfection. When the density reached approximately 1 × 10^6 ^cells/ml the cells were transfected. In 5 ml of unsupplemented FREESTYLE media 25 μg of plasmid DNA was added followed by 50 μg of the linear cationic polymer polyethylenimine (PEI) (Polysciences), prepared according to Durocher et al. [7], the mixture was vortexed, incubated at room temperature for 10 min and added to the cells. Cells were incubated at 37°C, 5 % CO_2_, 40 % humidity and agitated at 120 rpm. Supernatants were harvested 5 days post transfection (dpt) by centrifugation at 1942 × g, 5 min at 4°C. Protease inhibitor cocktail III (Novagen) was added to the cleared supernatant prior to storage at 4°C. Small scale transient transfections were performed where HEK-293E cells were seeded in 24 well blocks at either 0.5 × 10^6 ^cells/ml or 1 × 10^6 ^cells/ml in a total volume of 2 ml and pluronic added to a final concentration of 0.1 %. Cells at 0.5 × 10^6 ^cells/ml were transfected with DNA/PEI complex made by adding 0.5 μg of DNA to 200 μl of unsupplemented FREESTYLE media followed by 2 μg of linear PEI and treated as above. Cells at 1 × 10^6 ^cells/ml were transfected as above except 1 μg DNA was used. Blocks were incubated at 37°C, 5 % CO_2_, 40 % humidity, 400 rpm and supernatants were harvested 5 dpt as above.

### Western blot analysis

Secreted protein expression was analysed by adding 16.25 μl of supernatant to a 96-well plate containing 6.25 μl of 4 × NuPage LDS loading buffer (Invitrogen) and 2.5 μl of reducing agent (Invitrogen), the plate heated at 70°C for 10 min and 10 μl loaded onto a 17-well 4 – 12 % NuPAGE Bis-Tris gel (Invitrogen) using a multi-channel syringe (Hamilton). A His-tagged molecular weight marker was run on each gel (Qiagen). Proteins were electro-transferred onto a 0.45 μM PVDF membrane (Invitrogen). Membranes were blocked for 1 hr with 3 % low fat dried milk powder (Marvel) in phosphate buffered saline (PBS) – 0.1 % tween, incubated for 1 hr with anti-His5 monoclonal antibody (Novagen) at 40 ng/ml in 3 % Marvel-PBS-Tween, washed 3 × in PBS-Tween, incubated with 1 μg/ml of Cy5 labelled goat anti-mouse antibody (Amersham) in 3 % Marvel-PBS-Tween for 1 hr, washed 3× in PBS-Tween followed by a final wash in water prior to the blots being dried at 37°C for 10 mins between blotting paper. The blots were scanned on a Typhoon 8600 variable mode imager (Amersham) with fluorescence scan mode, 633 nm excitation laser, 670 nm emission filter, 600 V PMT and 200 μm/pixel scan resolution. The fluorescence intensity volumes of bands on the gel were quantitated using ImageQuant TL software (Amersham).

### Dot blot analysis

Purified proteins were diluted in mock supernatant prior to analysis on dot blot. Both purified proteins and test supernatants were denatured by addition of urea (to a final concentration of 5 M) at room temperature for 1 hour. Samples (300 μl/well) for dot blot analysis were loaded onto the Minifold I dot blot apparatus (Schleicher and Schuell) using a multi-channel pipette (Matrix) and allowed to adsorb to the protan nitrocellulose membrane (Whatman) under vacuum. Dot blot membranes were blocked and probed as described above for the western blots.

### Purification of 50 ml culture supernatants

Purification of His tagged proteins was performed using an AKTA 3D system (GE Healthcare). Supernatants were pre-conditioned by the addition of 4 M imidazole and 5 M NaCl to give final concentrations of 40 mM and 200 mM respectively. These were left at 4°C for 10 minutes and then spun at 27,750 g at 4°C for 10 min. The clarified supernatants were loaded sequentially at 1.5 ml/min onto five 1 ml Nickel Sepharose columns (GE Healthcare), pre-equilibrated with 2 × PBS (20 mM phosphate (pH7.4), 300 mM NaCl). The columns were then washed with 9.8 column volumes (CV) 2 × PBS each to remove non-specifically bound material. The second wash of the affinity columns was performed with 20 % B (400 mM imidazole, 500 mM NaCl, 50 mM Bicine pH 8.0) mixed in to increase the stringency (effectively 80 mM imidazole) of the wash. The columns were then washed back to 0 % B (2 × PBS) over 2CV prior to elution by step gradient to 100 % B. Eluted peaks were collected temporarily into a 5 ml loop prior to re-injection to a desalting column. The desalting column (2 × 5 ml HiTrap Desalt GE Healthcare) was pre-equilibrated with 50 mM sodium acetate, 20 mM NaCl pH 5.0 to exchange His tagged proteins into the running buffer of the Resource S cation exchange column (GE Healthcare) providing the final concentration step. The protein peak eluted from the desalting column was collected into a 10 ml loop prior to re-injection onto the Resource S column. Elution from the cation exchange column was carried out with a step gradient to 50 mM Bicine, 500 mM NaCl, 1.25 mM tris(hydroxypropyl)phosphine (THP). The protein was eluted in 1 ml and ready for quantitation.

### Quantitation of purified proteins

Quantitation of the His tagged proteins was done by analytical size exclusion chromatography (SEC). Proteins were first centrifuged at 25,000 g for 10 minutes to remove insoluble aggregates. The Fast Desalt PC3.2/10 (GE Healthcare) column was used directly connected to the flow cell on the AKTA Purifier 10 system (GE Healthcare) with an Auto-sampler. The column was equilibrated with 2 × PBS, 10 mM EDTA and run at 0.6 ml/min, 25 μl of each sample was injected onto the column. The peak area under the curve was converted to concentration in microgramme per milliliter using the calculated extinction coefficient for each protein [20,26].

## Authors' contributions

SC designed study, participated in the molecular biology, cell culture, transfections, performed the dot blot work, expression analysis and drafted the manuscript. AC participated in the cell culture, transfections and performed the western blot work. SPS performed protein purification of 50 ml transfection cultures. JM initiated work on the mammalian expression system and helped to draft manuscript and MD participated in the molecular biology, experimental design and helped to draft manuscript. All authors read and approved the final manuscript.

## Figures and Tables

**Table 1:** Expression vectors used in study

**Vector**	**Gene name**	**Ensemble ID**	**C-tag**	**Mwt (kDa)**
#1	Mfap3	ENSMUSP00000020830	rCD4-His10	36.99
#2	CLM9	ENSMUSP00000017453	rCD4-His10	39.97
#3	Undefined	ENSMUSP00000024791	rCD4-His10	33.43
#4	4732429D16Rik	ENSMUSP00000047022	rCD4-His10	41.51
#5	BC025105	ENSMUSP00000051521	rCD4-His10	42.77
#6	Fcer1a	ENSMUSP00000056882	rCD4-His10	44.67
#7	Cd2	ENSMUSP00000029456	rCD4-His10	45.15
#8	Tmem25^1^	ENSMUSP00000002100	rCD4-His10	47.03
#9	Tmem25^1^	ENSMUSP00000002100	rCD4-His10	47.03
#10	Tmem25^2^	ENSMUSP00000002100	rCD4-His10	47.03
#11	F11r	ENSMUSP00000041907	rCD4-His10	47.44
#12	Ptpns1	ENSMUSP00000049022	rCD4-His10	62.61
#13	Il18rap	ENSMUSP00000027237	rCD4-His10	63.21
#14	Unc5b	ENSMUSP00000077080	rCD4-His10	63.45
#15	Fgfrl1	ENSMUSP00000013633	rCD4-His10	62.41
#16	AI415330	ENSMUSP00000059913	rCD4-His10	66.44
#17	Ly9	ENSMUSP00000069319	rCD4-His10	72.27
#18	Scn3b	ENSMUSP00000051627	rCD4-His10	46.60
#19	E130306I01Rik	ENSMUSP00000061906	rCD4-His10	65.66
#20	Kit	ENSMUSP00000005815	rCD4-His10	94.62
#21	Lag3	ENSMUSP00000032217	rCD4-His10	69.63
#22	Lrrn1	ENSMUSP00000037096	rCD4-His10	92.52
Control rCD4	CD4	ENSRNOP00000021915	His10	24.20
Control EfnB2	EfnB2	ENSMUSP00000001319	His8	23.29
Control mCD4	CD4	ENSMUSP00000024044	His10	26.30
Control mCD200	CD200	ENSMUSP00000023341	rCD4-His10	46.57

Expression vectors (#1 – #22) encoding the extra-cellular region of mouse receptor genes used for deep well block transient expression study. Tmem25^1 ^contained a frameshift mutation and Tmem25^2 ^contained no mutations relative to the reference sequence. Included in this table are the four control vectors rat CD4 (domains 3 + 4), mouse EfnB2 (extra-cellular region), mouse CD4 (domains 3 + 4) and mouse CD200 (extra-cellular region).

**Table 2:** Expression analysis of proteins.

		**Fluorescence Intensity (×10^4^)**				
				
		**50 ml culture**	**2 ml culture**			
**Vector**	**Protein**	**Western blot**		**Dot blot**	**Ex-IMAC AKTA (μg)**	**Analytical SEC (μg)**

#1	Mfap3	0	0	0.4	0	-
#2	CLM9	850.6	657.1	298.4	535.5	124.6
#3	Undefined	1228.5	1054.3	1516.6	833.1	526.0
#4	4732429D16Rik	0	0	15.8	0	-
#5	BC025105	19.6	56.3	9.7	0	-
#6	Fcer1a	795.9	456.0	909.6	256.0	97.8
#7	Cd2	900.6	584.5	432.2	483.1	387.2
#8	Tmem25	0	0	5.6	0	-
#9	Tmem25	0	0	6.8	0	-
#10	Tmem25	53.6	122.3	147.5	117.9	-
#11	F11r	0	0	44.4	79.3	-
#12	Ptpns1	0	0	0.2	0	-
#13	Il18rap	65.4	65.3	152.0	70.3	-
#14	Unc5b	419.7	101.9	206.0	297.3	68.6
#15	Fgfrl1	0	0	5.0	64.6	-
#16	AI415330	312.1	58.3	1081.3	191.2	-
#17	Ly9	494.6	425.5	485.9	387.5	57.6
#18	Scn3b	0	0	5.6	0	-
#19	E130306I01Rik	0	0	25.3	0	-
#20	Kit	0	0	4.2	0	-
#21	Lag3	410.7	428.6	371.1	296.8	73.1
#22	Lrrn1	182.6	90.4	117.4	131.3	-

Fluorescent intensity of bands on western blot and dots on dot blots were analysed using ImageQuant software. The Ex-IMAC AKTA column values are calculated by integrating the elution peak from the sephadex column between the IMAC and ion-exchange steps. The Analytical SEC column values are calculated by analysing the purified proteins (see Methods section). There is a cut off during AKTA purification, if the imidazole strip peak detects < 200 ug the fraction is not collected and therefore no protein is purified and there is nothing to analyse on the SEC. All values are normalised to the same input volume of 50 ml for western blot, dot blot and purification analysis. Abbreviations: IMAC (Immobilised Metal Affinity Chromatography), SEC (Size Exclusion Chromatograpy).
